# Integrated metabolomics and gut microbiota analyses reveal the protective effects of matrine in ulcerative colitis

**DOI:** 10.3389/fchem.2026.1826894

**Published:** 2026-05-13

**Authors:** Linqi Su, Yu Zhang, Yixuan Xie, Jiazhen Wu, Yang Yang, Yun Li, Yongtong Huang, Xin Liu, Xuemei Wei, Qinhua Chen

**Affiliations:** 1 Shenzhen Traditional Chinese Medicine Hospital, The Fourth Clinical Medical College of Guangzhou University of Chinese Medicine, Shenzhen, China; 2 Key Laboratory of TCM Clinical Pharmacy, Shenzhen Baoan Authentic TCM Therapy Hospital, Guangzhou University of Chinese Medicine, Shenzhen, China

**Keywords:** internal transcribed spacer, matrine, shotgun metagenomic, ulcerative colitis, untargeted metabolomic analysis

## Abstract

**Background:**

Ulcerative colitis (UC) is a chronic inflammatory bowel disease driven by gut microbial dysbiosis and metabolic dysfunction. Matrine, a natural alkaloid with anti-inflammatory properties, shows therapeutic potential; however, its mechanisms involving the coordinated modulation of bacteria, fungi, and host intestinal luminal metabolism remain unclear.

**Methods:**

We evaluated the therapeutic efficacy of matrine using a dextran sulfate sodium (DSS)-induced murine model of ulcerative colitis. Disease severity was assessed via the disease activity index, colon length, and histopathology. Integrated multi-omics approaches, including metagenomics, ITS fungal sequencing, and untargeted metabolomics of intestinal luminal contents, were employed to systematically characterize the regulatory effects of matrine on gut bacteria, fungi, and metabolic profiles.

**Results:**

Here, we demonstrated that oral matrine significantly alleviated disease severity in a DSS-induced UC mouse model, as evidenced by improved disease activity index, colon length, histopathology, and restoration of tight junction proteins. Integrated multiomics revealed that matrine restored bacterial homeostasis—suppressing Escherichia while enriching SCFAs-producing taxa (Muribaculum, Paramuribaculum, Clostridium). Metagenomic predictions revealed that matrine treatment reversed the model-induced suppression of carbohydrate metabolism and bile acid biosynthesis while upregulating depleted CAZy enzyme families, thereby correcting dysregulated metabolic functions in colitis. Furthermore, matrine rebalanced the mycobiota by normalizing the Ascomycota/Basidiomycota ratio. Intestinal luminal contents untargeted metabolomics identified 43 matrine-responsive metabolites, implicating correction of bile acid metabolism, attenuation of leukotriene-mediated inflammation, and reversal of acylcarnitine-driven epithelial energy disruption. Critically, pro-inflammatory metabolites correlated positively with Escherichia and negatively with beneficial symbionts.

**Conclusion:**

Our findings established that matrine exerted protective effects in UC through a unified “microbiota–metabolism” axis, highlighting its promise as a multi-target therapeutic agent for UC.

## 1 Introduction

Ulcerative colitis (UC) is a chronic form of inflammatory bowel disease (IBD) characterized by persistent inflammation of the colonic mucosa (Adams and Bornemann, 2013). The global incidence of UC has been steadily rising, particularly in rapidly developing countries and regions, where shifts in lifestyle and increasing psychosocial stress have contributed to its emergence as a significant public health concern (Du L and Ha, 2020). Clinically, UC manifests as recurrent diarrhea, lower abdominal pain and cramping, hematochezia, and weight loss; severe cases may progress to life-threatening complications such as massive hemorrhage or colonic perforation (Voelker, 2024). Current therapeutic strategies primarily aim to control inflammation, alleviate symptoms, and prevent relapse, relying on agents including 5-aminosalicylic acid (5-ASA), corticosteroids (e.g., prednisone), immunosuppressants (e.g., azathioprine), and biologics (e.g., infliximab) (Segal et al., 2021). However, these treatments are often associated with considerable adverse effects, and a subset of patients exhibits poor response or develops tolerance to conventional therapies. Consequently, the discovery of novel, efficacious, and low-toxicity therapeutics from natural products has become a major focus in UC drug development.

Accumulating evidence highlights a pivotal role for gut microbiota dysbiosis in the pathogenesis of UC (Liu B. et al., 2022). Patients with UC commonly exhibit an imbalanced gut microbial ecosystem—marked by depletion of beneficial taxa and expansion of potential pathobionts—which compromises intestinal barrier integrity, facilitates microbial translocation, and exacerbates mucosal inflammation (Guo et al., 2020). While most studies have concentrated on bacterial alterations, gut fungi, as integral components of the gut mycobiota, are increasingly recognized for their contribution to UC pathology. Notably, UC patients display significantly elevated fungal diversity compared to healthy individuals, disrupting the homeostatic interplay between bacteria and fungi and thereby impairing barrier function (Wang H. et al., 2023). Specific fungal overgrowth, such as *Candida* species, can directly potentiate inflammation (Balderramo et al., 2023). Moreover, the bidirectional interaction between gut fungi and the host immune system is crucial for maintaining intestinal homeostasis (Kong et al., 2024). Conversely, the inflamed colonic environment in IBD promotes fungal colonization; for instance, mucosal fungal burden is higher in Crohn’s disease patients than in healthy controls (McCrory et al., 2024). Antibiotic use during IBD flares—a common clinical practice—further favors fungal proliferation, as antibiotics suppress bacterial competitors. Certain fungi can colonize ulcerated lesions, interfere with wound-healing signaling pathways, and perpetuate inflammation (Chiaro and Round, 2021). Despite these insights, research into the precise roles and regulatory mechanisms of gut fungi in colitis remains limited, leaving a critical gap in our holistic understanding of the gut microbiome.

Gut microbes exert profound influence on host physiology largely through their bioactive metabolites, including short-chain fatty acids (SCFAs), bile acids (BAs), and tryptophan derivatives, which modulate local and systemic immune and metabolic homeostasis. Dysregulation of these metabolic axes is tightly linked to the inflammatory state in UC (Wang J. et al., 2023), making the “gut microbiota–host metabolism” axis a Frontier in mechanistic and therapeutic research (Liu et al., 2024). SCFAs produced by commensal bacteria through dietary fiber fermentation, attenuate inflammation by inhibiting histone deacetylases and activating G protein–coupled receptors to promote the differentiation of anti-inflammatory regulatory T cells and IL-10–producing B cells (Mukhopadhya and Louis, 2025). UC patients typically exhibit markedly reduced SCFAs levels, contributing to barrier dysfunction and uncontrolled inflammation. Gut bacteria convert primary BAs into secondary BAs via bile salt hydrolases and 7α-dehydroxylase. These secondary BAs suppress NLRP3 inflammasome activation by acting as ligands for TGR5—triggering the cAMP–PKA pathway to promote NLRP3 phosphorylation and ubiquitination—and inhibit IL-1β/IL-18 release (Debnath et al., 2021). Disruption of microbial BAs metabolism thereby exacerbates colonic inflammation. Similarly, microbial catabolism of tryptophan generates indole derivatives that activate the aryl hydrocarbon receptor (AhR) in intestinal epithelial and immune cells, driving IL-22–mediated barrier fortification, antimicrobial immunity, and Treg differentiation—collectively exerting anti-inflammatory and protective effects in ulcerative colitis (Su et al., 2022). Untargeted metabolomics, as a cornerstone of systems biology, enables comprehensive, unbiased profiling of dynamic metabolic changes in biological systems, making it indispensable for identifying disease biomarkers and elucidating drug mechanisms (Ge et al., 2025; Liao et al., 2019). Integrating metabolomic data with microbiome analyses allows for the reconstruction of intricate “microbe–metabolite–host” interaction networks, offering deep mechanistic insights into pharmacological interventions (Lv et al., 2024; Zhang et al., 2024).

Matrine, a tetracyclic quinolizidine alkaloid extracted from *Sophora flavescens* Aiton and other leguminous plants, exhibits potent anti-inflammatory, antioxidant, immunomodulatory, and antitumor activities (Lin et al., 2022). Previous studies have demonstrated that matrine alleviated colitis by enhancing intestinal barrier integrity, inhibiting the PPAR-α signaling pathway, and modulating gut bacterial composition (Li et al., 2019; Mao et al., 2024; Yao et al., 2021). However, existing investigations into its microbiota-modulating effects have largely relied on 16S rRNA gene sequencing, which lacks species-level resolution and completely overlooks the fungal community (Dou et al., 2024). Moreover, the systemic mechanisms by which matrine orchestrates specific microbial taxa and their associated metabolic networks to confer protection against UC remain unexplored through integrated multi-omics approaches.

Therefore, in this study, we first evaluated the therapeutic efficacy of matrine in a dextran sulfate sodium (DSS)-induced murine colitis model. We then employed shotgun metagenomic sequencing and internal transcribed spacer (ITS) sequencing to comprehensively characterized the remodeling of colonic bacterial and fungal communities, respectively. Concurrently, untargeted metabolomics was applied to identify key metabolites altered by matrine treatment. Finally, through multi-omics correlation analysis, we aimed to decipher the regulatory network linking “matrine–functional microbes–core metabolites”. This work provided robust experimental and theoretical support for the development of matrine as a promising candidate for UC therapy and offered a novel perspective on how herbal monomers modulate the “microbiota–metabolism” axis.

## 2 Materials and methods

### 2.1 Chemicals and reagents

Dextran sulfate sodium (DSS; molecular weight 36,000–50,000) and 5-aminosalicylic acid (5-ASA) were obtained from MeilunBio Technology Co., Ltd. (Dalian, China). Matrine (purity >98%) was purchased from Shanghai Yuanye Bio-Technology Co., Ltd. (Shanghai, China). Carboxymethyl cellulose sodium salt (CMC-Na) and formic acid were acquired from Shanghai Macklin Biochemical Co., Ltd. (Shanghai, China). Acetonitrile and methanol were supplied by Merck KGaA (Darmstadt, Germany). 4% paraformaldehyde solution, hematoxylin, solvent red 43 and neutral balsam were provided by Beijing Solarbio Science & Technology Co., Ltd. (Beijing, China). Bovine Serum Albumin (BSA), Anti-Occludin Rabbit pAb, Anti-Claudin Rabbit pAb and DAPI were purchased from Wuhan Servicebio Technology Co., Ltd. (Wuhan, China).

Stool genomic DNA was extracted using the FastPure Stool DNA Isolation Kit (Magnetic Bead) (MJYH Biotech, Shanghai, China). Agarose (Cat. No. 75510019) was purchased from Thermo Fisher Scientific Inc. (Waltham, MA, USA). Library preparation was performed with the NEXTFLEX® Rapid DNA-Seq Kit (Bioo Scientific, Austin, TX, USA). Sequencing reagents were provided by the DNBSEQ™-T7 RS Reagent Kit (FCL PE150), Version 3.0 (BGI Genomics Co., Ltd., Shenzhen, China).

### 2.2 Animal experiments

#### 2.2.1 Animal models and experimental grouping

Twenty-four male C57BL/6J mice (8 weeks old, weighing 20 ± 2 g) were purchased from the Guangdong Provincial Laboratory Animal Center. All animals were housed under controlled environmental conditions (22 °C–24 °C, 50%–60% humidity, 12-h light/dark cycle) with *ad libitum* access to standard chow and water. All operations involving animal experiments followed the relevant requirements of the Institutional Animal Care and Use Committee (TOP-IACUC-2024-0319) of Shenzhen TOP Biotechnology Co., Ltd. After a 7-day acclimatization period, the mice were randomly assigned into four groups (n = 6 per group) based on body weight: Control group (Control), DSS-induced colitis model group (Model), positive control group treated with 5-ASA (5-ASA), and matrine-treated group (Matrine). Based on the literature research (Mao et al., 2024), we selected 20 mg/kg as the dosage for the administration of matrine.

**FIGURE 1:**
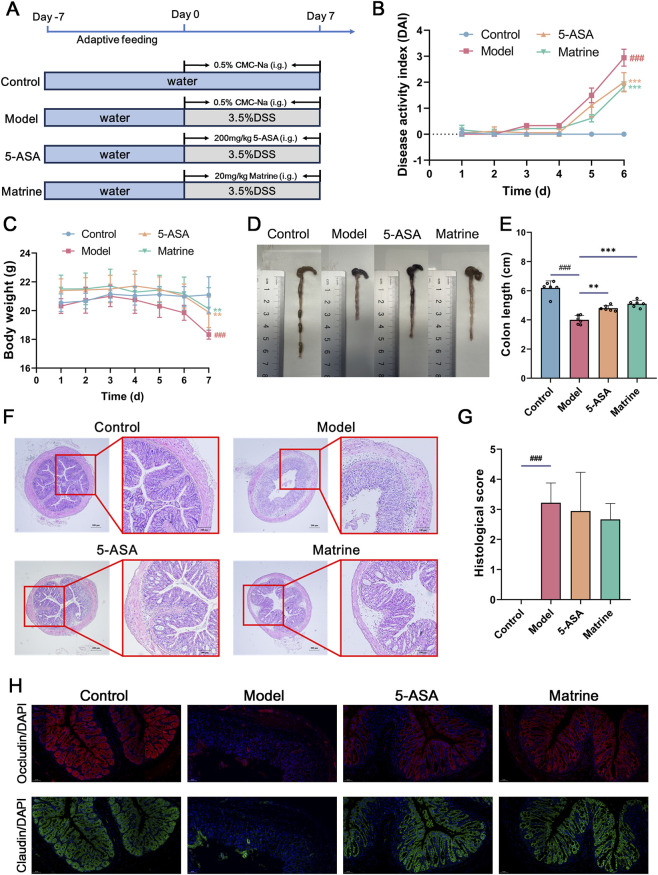
Evaluation of the therapeutic effects of matrine on DSS-induced ulcerative colitis (UC) in mice and its impact on intestinal barrier integrity. **(A)** Schematic illustration of the experimental timeline. **(B)** Body weight changes over time in each group. **(C)** DAI scores throughout the experimental period. **(D)** Representative images of colons from each group. **(E)** Quantification of colon length. **(F)** Representative H&E-stained histological sections of colonic tissue. **(G)** Histopathological scoring of colonic inflammation. **(H)** Immunofluorescence staining of occludin and claudin in colonic tissue. Statistical significance: compared with the Control group, ^#^ *P* < 0.05, ^##^ *P* < 0.01, ^###^ *P* < 0.001; compared with the Model group, ^*^ *P* < 0.05, ^**^ *P* < 0.01, ^***^ *P* < 0.001.

The experimental design is illustrated in Figure 1A. Mice in the control group received normal drinking water, whereas those in the model, 5-ASA, and matrine groups were administered a 3.5% DSS solution to induce UC. During the experimental period, the control and model groups were treated with 0.5% CMC-Na solution via oral gavage. In contrast, the 5-ASA and matrine groups received oral gavage of 5-ASA (200 mg/kg) dissolved in 0.5% CMC-Na and matrine (20 mg/kg), respectively. Body weight and general health status were monitored daily during the experimental period. After the final dose and following a 12-h fasting period with free access to water, all mice were anesthetized. Blood was collected via retro-orbital puncture, and the entire colon was carefully excised. Colon length was measured and documented photographically. A segment of the distal colon was immediately fixed in 4% paraformaldehyde for histological analysis, while luminal contents were rapidly snap-frozen in liquid nitrogen and stored at −80 °C for subsequent microbiota and metabolomic analyses.

#### 2.2.2 Measurement of disease activity index (DAI) score

During the modeling period, body weight changes, stool consistency, and presence of fecal blood were monitored daily in all mice, and a DAI score was calculated for each animal according to the criteria outlined in Supplementary Table S1. The DAI was determined as the arithmetic mean of three parameters: weight loss, stool consistency, and fecal bleeding, using the following formula:

$$\text{DAI}=\left(\text{Score for weight loss}+\text{Score for stool consistency}+S\text{core for fecal bleed}\right)\;/3$$

#### 2.2.3 Histological analysis

Distal colonic segments were harvested and immediately fixed in 4% paraformaldehyde, followed by paraffin embedding and sectioning. Tissue sections were subjected to hematoxylin and eosin (H&E) staining and examined under a light microscope to evaluate histopathological alterations in the colon across experimental groups. Representative photomicrographs were captured and documented. Histopathological injury was scored independently by two blinded observers according to the criteria detailed in Supplementary Table S2.

For immunofluorescence staining, after deparaffinization, rehydration, and citrate antigen retrieval, sections were blocked with 3% BSA and subjected to sequential tyramide signal amplification (TSA)-based dual staining for rabbit anti-occludin and anti-claudin antibodies, using Fluorophore 555 and 488, respectively, with antibody stripping between steps. Nuclei were counterstained with DAPI, autofluorescence was quenched, and slides were imaged on a Nikon Eclipse C1 confocal microscope.

### 2.3 Gut microbiota shotgun metagenomic sequencing analysis

The gut microbial communities in mouse intestinal contents were analyzed using shotgun metagenomic sequencing, with technical support provided by Majorbio Bio-Pharm Technology Co., Ltd. (Shanghai, China). 0.2g of stool material was used to extract total genomic DNA with the FastPure® Stool DNA Isolation Kit (Magnetic Bead) (MJYH Biotech, Shanghai, China) according to manufacturer’s instructions. Concentration and purity of extracted DNA was determined with Synergy HTX (Biotek, USA) and NanoDrop2000 (Thermo Scientific Inc., USA), respectively. DNA quality was checked on 1% agarose gel. Detailed experimental procedures are provided in the Supplementary Material.

### 2.4 Internal transcribed spacer (ITS) sequencing analysis

Total microbial genomic DNA was extracted from intestinal luminal contents of mice using the FastPure Stool DNA Isolation Kit (MJYH, shanghai, China) according to manufacturer’s instructions. The quality and concentration of DNA were determined by 1.0% agarose gel electrophoresis and a NanoDrop®ND-2000 spectrophotometer (Thermo Scientific Inc., USA) and kept at 80 °C prior to further use. The fungal ITS1–ITS2 region was amplified with the primer pair ITS1F (5′-CTT​GGT​CAT​TTA​GAG​GAA​GTA​A-3′) and ITS2R (5′-GCT​GCG​TTC​TTC​ATC​GAT​GC-3′) using a T100 Thermal Cycler (Bio-Rad, USA). Detailed experimental procedures are provided in the Supplementary Material.

### 2.5 Untargeted metabolomic analysis of intestinal luminal contents

Chromatographic separation was performed on an Agilent 1290 II UHPLC system (Agilent Technologies, Santa Clara, CA, USA) equipped with a ZORBAX Eclipse XDB-C18 column (2.1 × 100 mm, 1.8 μm). Detailed sample processing, instrument parameter settings, and data acquisition and analysis can be found in the Supplementary Material.

Raw data were processed using Progenesis QI (Waters Corporation) for peak detection, alignment, and metabolite annotation. Putative metabolites were subsequently identified through spectral matching against MS-DIAL and the Human Metabolome Database (HMDB).

### 2.6 Statistical analysis

Statistical analyses were performed using GraphPad Prism 8 (GraphPad Software, San Diego, CA, USA). Data are presented as mean ± standard deviation ( $\bar{x}\pm s$ ). Comparisons among multiple groups were conducted by one-way analysis of variance (ANOVA), followed by appropriate *post hoc* tests. A *P* value < 0.05 was considered statistically significant. Untargeted metabolomics data were processed and analyzed using the MetaboAnalyst 6.0 online platform (https://www.metaboanalyst.ca/) for comprehensive metabolomic profiling and statistical interpretation. Associations between microbial taxa and metabolites were assessed using Spearman’s correlation. Only strong (∣r∣>0.6) and significant (*P* < 0.05) correlations were considered for further interpretation.

## 3 Results and discussion

### 3.1 Matrine ameliorated DSS-induced UC symptoms in mice and effectively restored intestinal barrier function

Compared with the Control group, mice in the DSS-induced Model group exhibited pronounced disease-associated phenotypes during the modeling period. Body weight began to decline persistently from day 3 post-DSS administration and was markedly reduced by the end of the experiment (*P* < 0.001). The DAI score progressively increased with disease progression, reflecting hallmark clinical symptoms of colitis, including diarrhea and fecal occult/gross bleeding. Additionally, colon length in the Model group was significantly shortened (*P* < 0.001), indicative of intestinal inflammation–induced tissue edema, structural disruption, and functional impairment (Figures 1B–E).

In contrast, treatment with either 5-ASA or matrine ameliorated these clinical manifestations to varying degrees. Specifically, both the 5-ASA and Matrine groups showed a markedly attenuated rate of body weight loss (Figure 1B). Moreover, their DAI scores were significantly lower than those of the Model group (*P* < 0.001; Figure 1C), demonstrating effective alleviation of UC-related clinical symptoms. Colon length was also significantly restored in both treatment groups compared to the Model group (*P* < 0.001; Figures 1D,E), suggesting a protective effect on colonic architecture. Notably, matrine exhibited superior therapeutic efficacy compared to 5-ASA.

Histopathological analysis further corroborated these findings. H&E staining revealed that colonic mucosa in the Control group was intact, with tightly arranged epithelial cells, well-organized and regularly shaped villi, clearly defined tubular crypts, and abundant, uniformly distributed goblet cells. In contrast, the Model group displayed characteristic inflammatory damage: severe crypt distortion or complete loss, marked reduction in goblet cell numbers, and extensive infiltration of inflammatory cells in the lamina propria and submucosa (Figure 1F). Remarkably, mice treated with matrine exhibited substantially milder histopathological alterations—partial restoration of crypt architecture, increased goblet cell counts, and significantly reduced inflammatory cell infiltration compared to the Model group (Figure 1F). Histopathological scoring of colonic sections confirmed these observations: the Model group exhibited a dramatically elevated pathology score (*P* < 0.001), whereas the Matrine group showed a significant reduction in this score (Figure 1G). Collectively, these results indicate that matrine promotes colonic tissue repair and suppresses local inflammatory responses in DSS-induced colitis.

Tight Junction Proteins (TJPs) are a class of transmembrane protein complexes localized at the apical lateral borders of adjacent colonic epithelial cells, forming a continuous “molecular seal” that is essential for maintaining the structural integrity and selective permeability of the intestinal epithelial barrier (Liu X. et al., 2022). Among these, occludin and claudin are two canonical TJPs whose expression levels serve as critical indicators for assessing intestinal barrier integrity and disease activity in UC (Górecka et al., 2024). During therapeutic intervention, the expression of these TJPs in colonic tissue provides a reliable readout of epithelial barrier function and the severity of UC. Immunofluorescence staining of mouse colonic sections revealed that occludin and claudin were uniformly distributed and tightly localized along the epithelial cell borders in the Control group. In contrast, their expression was markedly diminished or discontinuous in the colonic epithelium of the Model group, indicative of severe barrier disruption. Notably, matrine treatment significantly restored the expression and proper localization of both occludin and claudin (Figure 1H), suggesting that matrine enhances intestinal barrier integrity by upregulating key TJPs.

In summary, the DSS-induced murine colitis model successfully recapitulated key pathological features of inflammatory bowel disease. Matrine intervention demonstrated considerable therapeutic potential by effectively alleviating clinical symptoms, restoring colon length, attenuating histopathological damage, and reinforcing intestinal barrier function.

### 3.2 Matrine alleviated DSS-induced UC in mice by modulating the gut microbiota

#### 3.2.1 Analysis of the modulatory effects of matrine on the intestinal bacterial community in UC mice

**FIGURE 2:**
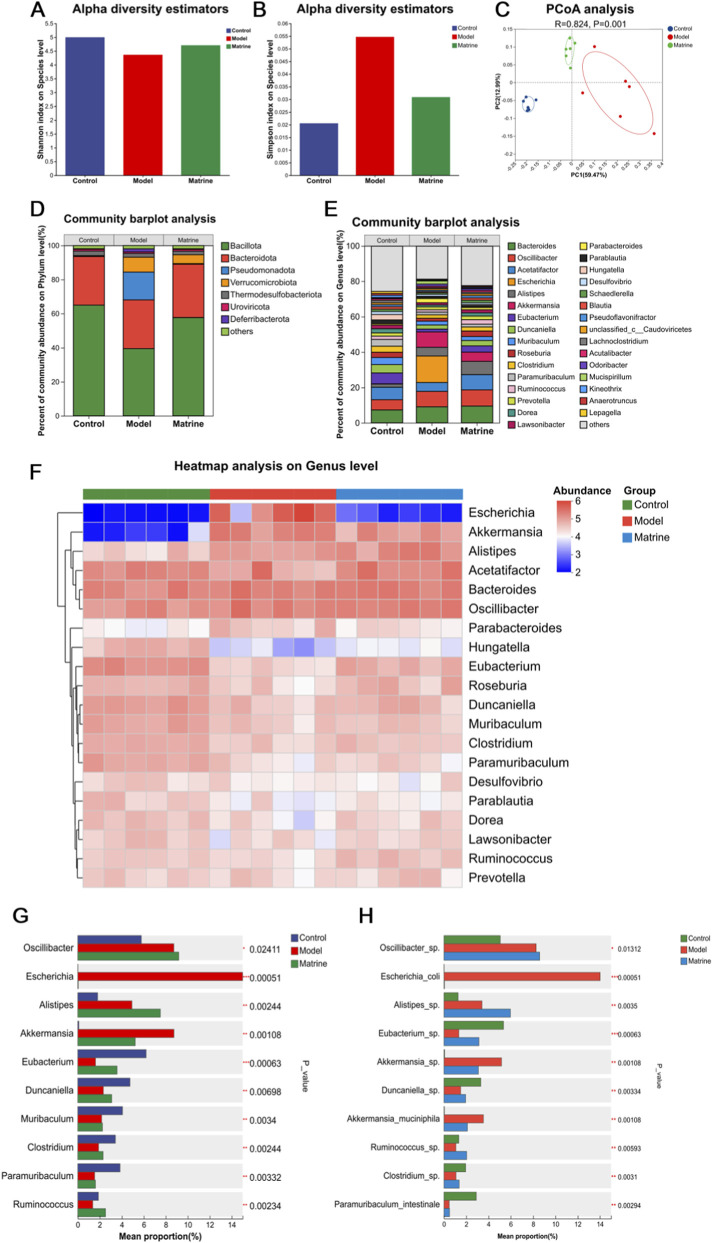
Alterations in diversity and composition of gut microbiota in DSS-treated mice with matrine administration. **(A)** Shannon diversity index. **(B)** Simpson diversity index. **(C)** PCoA score plot. **(D–F)** Relative abundance of gut bacterial communities at the phylum **(D)** and genus **(E)** levels. **(F)** Heatmap of the top 20 most abundant genera, depicting microbial community composition at the genus level. **(G,H)** Bar plot showing statistically significant differences in multiple bacterial taxa at the genus **(G)** and species **(H)** levels across groups. Statistical significance was determined using Kruskal-Wallis H test for multiple-group comparisons. ^*^ *P* < 0.05, ^**^ *P* < 0.01, ^***^ *P* < 0.001.

Gut microbiota dysbiosis is a well-recognized contributor to the pathogenesis of UC. To investigate the modulatory effects of matrine on the gut microbial community, we performed shotgun metagenomic sequencing of intestinal luminal contents from UC mice. α-diversity analysis revealed that the bacterial community diversity—assessed by Shannon and Simpson indices—was markedly reduced in the Model group compared to the Control group, whereas matrine treatment significantly restored microbial diversity (*P* < 0.05; Figures 2A,B). Principal Coordinates Analysis (PCoA) based on Bray–Curtis dissimilarity clearly separated the microbial profiles of the Matrine group from those of the Model group, indicating a distinct restructuring of the gut microbiota following matrine intervention (Figure 2C).

**FIGURE 3:**
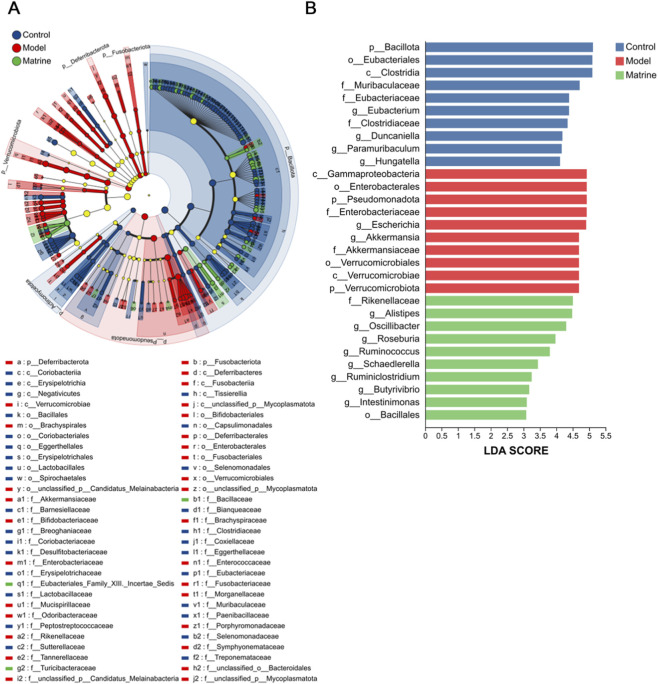
Identification of the most differentially abundant taxonomic features in gut microbiota across experimental groups and multi-group comparison analysis. **(A)** LEfSe cladogram illustrating taxonomic differences among groups. **(B)** LDA score histogram from LEfSe analysis identifying significantly enriched taxa.

Taxonomic composition analysis further elucidated the regulatory effects of matrine across multiple taxonomic levels. At the phylum level, matrine partially restored microbial balance in colitic mice, characterized by an increased relative abundance of *Bacillota* and a concomitant reduction in *Pseudomonadota* (Figure 2D). At the genus level, matrine exerted therapeutic effects by enriching *Acetatifactor*, *Eubacterium*, *Duncaniella*, and concurrently reducing the abundance of *Escherichia* (Figure 2E). Linear discriminant analysis Effect Size (LEfSe) was employed to identify differentially enriched taxa specific to each group. This analysis revealed that the Matrine group was significantly enriched in several beneficial or butyrate-producing taxa, including *f_Rikenellaceae*, *g_Alistipes*, *g_Oscillibacter*, *g_Roseburia*, *g_Ruminococcus*, *g_Schaedlerella*, *g_Ruminiclostridium*, *g_Butyrivibrio*, *g_Intestinimonas*, and *o_Bacillales* (Figure 3). Comparative analysis across all groups demonstrated that matrine effectively reversed DSS-induced dysbiosis by significantly modulating key genera, including *Escherichia*, *Akkermansia*, *Eubacterium*, *Duncaniella*, *Ruminococcus*, and *Clostridium*, as well as specific species such as *Escherichia coli* and *Akkermansia muciniphila*, with statistically significant differences observed between groups (Figures 2G,H). The comparison of the abundance of each group of bacterial species is shown in the Supplementary Figure S1. Specifically, matrine treatment significantly suppressed the DSS-induced overgrowth of *Escherichia coli*, while concurrently restoring the abundance of beneficial genera and species (e.g., *Eubacterium*, *Duncaniella*, and *Ruminococcus*) to levels approaching those of the Control group, thereby ameliorating the overall microbial imbalance. Furthermore, heatmap clustering confirmed that matrine treatment reshaped the microbial community by upregulating beneficial genera such as *Roseburia*, *Eubacterium*, *Duncaniella*, and *Hungatella*, while downregulating pathobionts like *Mucispirillum* and *Escherichia* (Figure 2F).

**FIGURE 4:**
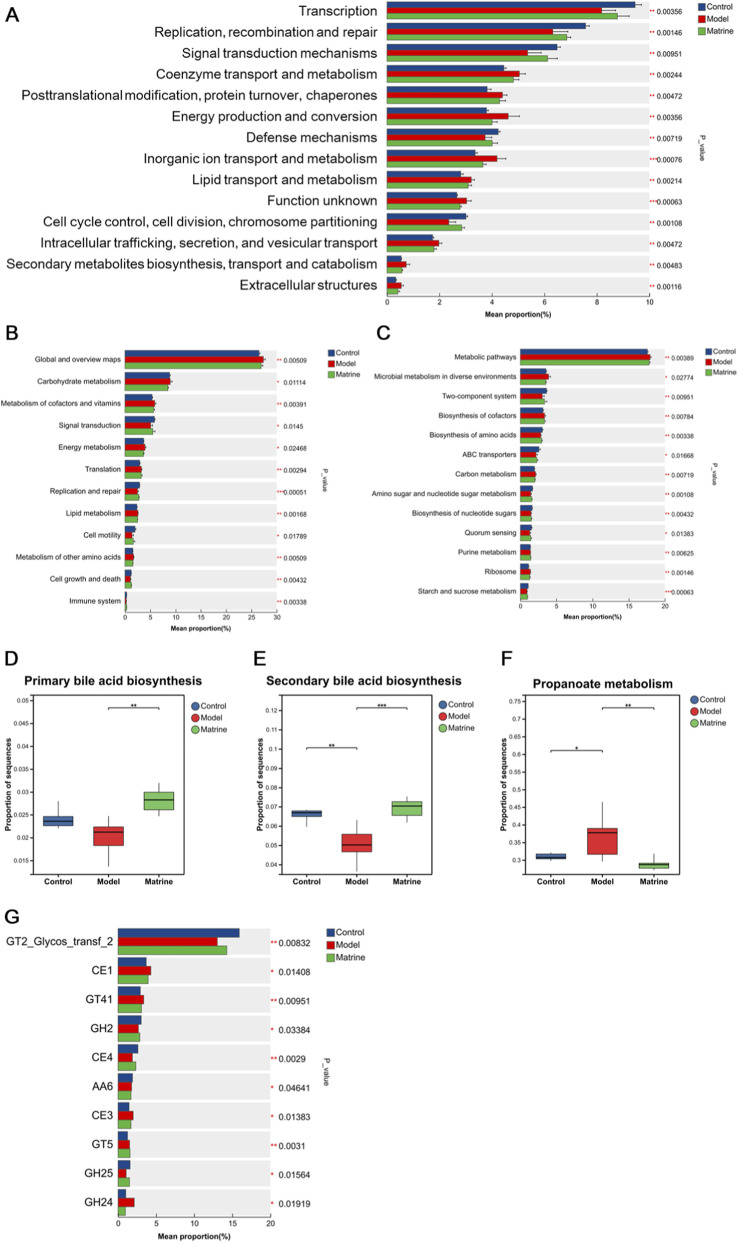
Functional alterations of the gut microbiome among the Control, Model, and Matrine groups. **(A)** Histogram of COG functional categories showing significant differences in relative abundance. **(B,C)** Bar plots of KEGG pathways at Level 2 **(B)** and Level 3 **(C)** based on Kruskal-Wallis H tests, highlighting metabolic pathways with significant variations. **(D–F)** Box plots illustrating the relative abundance of specific metabolic pathways: Primary bile acid biosynthesis **(D)**, Secondary bile acid biosynthesis **(E)**, and Propanoate metabolism **(F,G)** Relative abundance of significantly different CAZyme families (Carbohydrate-Active Enzymes) across the three groups. Abbreviations: GT, Glycosyltransferases; GH, Glycoside Hydrolases; CE, Carbohydrate Esterases; AA, Auxiliary Activities. Data are presented as mean ± SD (for bar plots) or median with interquartile range (for box plots). Asterisks indicate statistical significance (^*^ *P* < 0.05, ^**^ *P* < 0.01, ^***^ *P* < 0.001).

To investigate the functional consequences of these taxonomic shifts, we performed a metagenomic functional prediction analysis (Figure 4). COG functional classification showed that the Model group had significantly reduced relative abundances in categories such as *Transcription* and *Replication*, *recombination and repair* compared to the Control group, which were reversed by matrine treatment (Figure 4A). KEGG pathway analysis at Levels 2 and 3 revealed that metabolic pathways, including *Carbohydrate metabolism*, were generally suppressed in the Model group but restored by matrine (Figures 4B,C). Notably, while *Primary bile acid biosynthesis* and *Secondary bile acid biosynthesis* pathways were significantly downregulated in the Model group (P < 0.01), the *Propanoate metabolism* pathway showed a significant increase (P < 0.01), both of which were normalized by matrine treatment (Figures 4D–F). Additionally, CAZy family analysis indicated that matrine significantly upregulated key enzyme families (e.g., GT2, GH2, CE4) that were depleted in the Model group (Figure 4G). These results suggested that matrine ameliorates colitis not only by restructuring microbial composition but also by correcting dysregulated metabolic functions.

Studies have shown that in patients with UC, gut microbial dysbiosis is characterized by a significant reduction in the abundance of *Bacillota*—particularly a depletion of *Clostridium*-related clusters—which impairs SCFAs production and compromises intestinal barrier integrity. Concurrently, there is a marked expansion of *Pseudomonadota*, especially members of the *Enterobacteriaceae* family, whose overgrowth is positively correlated with elevated levels of pro-inflammatory cytokines and increased disease activity (Pisani et al., 2022). These microbial changes collectively contribute to the pathogenesis of UC, and this study found that matrine can effectively reverse these microbial dysfunctions.

It has been widely reported that *Escherichia*, a genus within *Enterobacteriaceae*, is frequently associated with various intestinal disorders and exhibits global distribution. Among its members, *Escherichia coli* is of particular concern due to its diverse pathogenic strains. Typical enteropathogenic *E. coli* (tEPEC) is a well-established primary cause of infantile diarrhea, while atypical EPEC (aEPEC) can induce a spectrum of symptoms ranging from mild to hemorrhagic diarrhea and may even serve as a precursor to Shiga toxin–producing *E. coli* (STEC) (Brook and Bannister, 1993; Hu and Torres, 2015). In the present study, matrine significantly suppressed the DSS-induced overgrowth of *Escherichia*, suggesting that inhibition of this pro-inflammatory pathobiont constitutes a key mechanism underlying matrine’s therapeutic efficacy in UC.

Conversely, beneficial taxa were restored by matrine treatment. *Eubacterium*, known for its critical roles in modulating intestinal immunity, suppressing inflammation, and participating in butyrate production and bile acid metabolism (Mukherjee et al., 2020), was markedly depleted in UC mice but effectively replenished following matrine administration. Similarly, *Duncaniella*—a genus recently shown to exert protective effects in DSS-induced colitis by enhancing epithelial barrier function and attenuating colonic injury (Chang et al., 2021)—was significantly downregulated in the Model group but restored to near-normal levels by matrine. Moreover, specific strains within *Clostridium* are recognized for their capacity to produce butyrate, reinforce the intestinal barrier, and mitigate inflammatory responses, thereby ameliorating acute DSS-induced colitis in preclinical models (Hua et al., 2025; Krause et al., 2024; Ma et al., 2022). Matrine treatment significantly increased the relative abundance of *Clostridium*, further supporting its role in promoting a favorable microbial environment.

In summary, the therapeutic effect of matrine in DSS-induced UC involves a comprehensive restoration of gut microbial homeostasis. Integrated analysis indicates that matrine acts through a dual regulatory mechanism: it suppresses pathobionts like *Escherichia* while promoting beneficial taxa such as *Eubacterium* and *Duncaniella*. Crucially, this taxonomic restructuring is accompanied by the functional correction of microbial metabolism, specifically the normalization of bile acid and propanoate metabolism pathways. This rebalancing of both microbial composition and metabolic function likely underpins matrine’s anti-inflammatory and barrier-protective actions in UC.

#### 3.2.2 Analysis of the modulatory effects of matrine on intestinal fungi in UC mice

**FIGURE 5:**
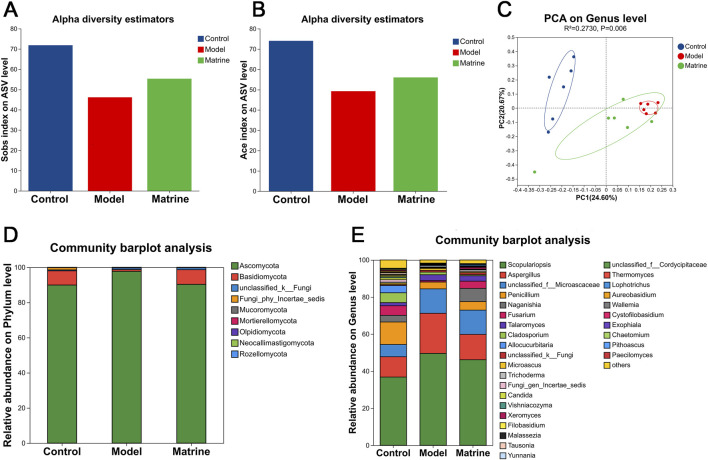
Matrine alleviates DSS-induced ulcerative colitis in mice by modulating the gut fungi. **(A) **Sobs index. **(B)** Ace index. **(C)** PCA score plot. **(D,E)** Relative abundance of gut fungal communities at the phylum **(D)** and genus **(E)** levels.

To assess the impact of matrine on the intestinal mycobiota, ITS sequencing was conducted to characterize alterations in fungal community composition. α-diversity analysis of intestinal fungal communities in luminal contents revealed that the Model group exhibited significantly reduced richness, as indicated by lower observed species (Sobs) and Abundance-based Coverage Estimator (Ace) indices compared to the Control group, whereas matrine treatment partially restored fungal diversity (Figures 5A,B). β-diversity analysis was performed to assess intergroup dissimilarities in fungal composition. Principal Component Analysis (PCA) revealed a clear separation between the Control and Model groups, while the Matrine group exhibited a distinct shift away from the Model group toward the Control group, although complete segregation was not achieved, indicating that matrine partially ameliorated DSS-induced perturbations of the gut fungal community (Figure 5C).

**FIGURE 6:**
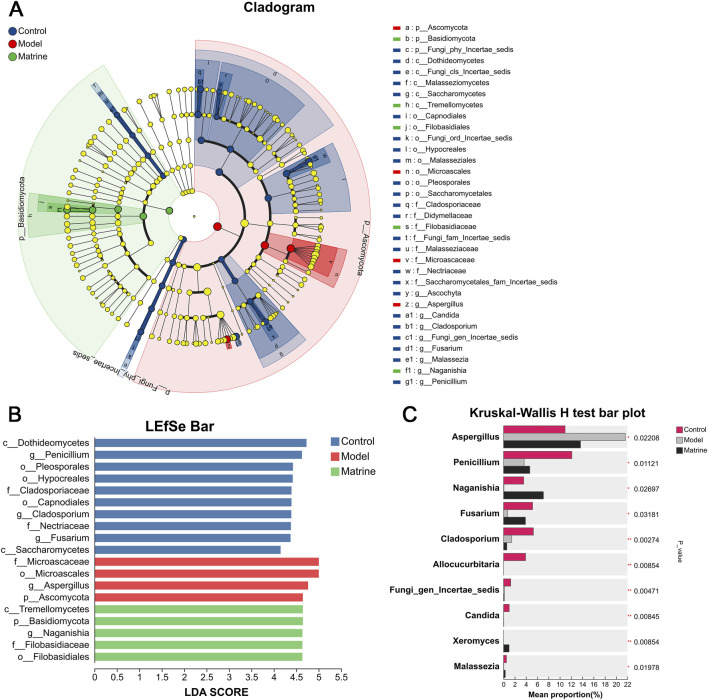
Identification of the most differentially abundant taxonomic features in gut fungi across experimental groups and multi-group comparison analysis. **(A)** LEfSe cladogram illustrating taxonomic differences among groups. **(B)** LDA score histogram from LEfSe analysis identifying significantly enriched taxa. **(C)** Bar plot showing statistically significant differences in multiple fungal taxa across groups. Statistical significance was determined using Kruskal-Wallis H test for multiple-group comparisons. ^*^ *P* < 0.05, ^**^ *P* < 0.01, ^***^ *P* < 0.001.

Taxonomic profiling further delineated the modulatory effects of matrine on the mycobiota across multiple taxonomic levels. At the phylum level, matrine rebalanced the fungal community by decreasing the relative abundance of *Ascomycota* and increasing that of *Basidiomycota* (Figure 5D). Consistently, genus-level analysis revealed that matrine increased the abundance of *Naganishia* and *Fusarium*, and markedly suppressed *Scopulariopsis* and *Aspergillus* (Figure 5E). To further identify group-specific fungal signatures, LEfSe was employed to identify group-specific fungal biomarkers, using an LDA score threshold >2 to define significantly differentially enriched taxa. The Model group was uniquely enriched in *f_Microascaceae*, *o_Microascales*, *g_Aspergillus*, and *p_Ascomycota*, all of which are associated with dysbiosis and inflammation. In contrast, the Matrine group showed specific enrichment of beneficial or commensal-related taxa, including *c_Tremellomycetes*, *p_Basidiomycota*, *g_Naganishia*, *f_Filobasidiaceae*, and *o_Filobasidiales* (Figures 6A,B). Furthermore, multi-group comparative analysis identified several fungal genera with statistically significant differences among the three groups. Notably, matrine exerted a pronounced restorative effect on the abundances of *Aspergillus*, *Penicillium*, *Naganishia*, and *Fusarium* at the genus level, effectively reversing DSS-induced fungal dysbiosis (Figure 6C).

α-diversity analysis revealed that DSS-induced UC markedly reduced the richness of the intestinal mycobiota in luminal contents, whereas matrine treatment effectively restored fungal richness. PCA further demonstrated that matrine ameliorated the DSS-induced perturbation of the gut fungal community, shifting its composition toward that of healthy controls.

Notably, previous studies have proposed the *Basidiomycota*/*Ascomycota* ratio—termed the “fungal dysbiosis index”—as a biomarker of gut mycobiota homeostasis in humans. This ratio is significantly elevated in patients with inflammatory bowel disease (IBD), reflecting fungal dysbiosis (Yadav et al., 2024). In contrast, in the DSS-induced murine UC model, the opposite pattern was observed: a significant increase in the relative abundance of *Ascomycota* and a concomitant decrease in *Basidiomycota* (Qiu et al., 2015). Our study demonstrates that matrine effectively reverses this imbalance in UC mice by decreasing *Ascomycota* and increasing *Basidiomycota*, thereby normalizing the fungal dysbiosis index. Comprehensive analysis of fungal community structure and intergroup differences across taxonomic levels (phylum, family, and genus) revealed that the mycobiota composition and relative abundances in the Matrine group closely resembled those of the Control group and were distinctly different from those of the Model group. Collectively, these findings indicate that DSS-induced colitis profoundly disrupts the equilibrium of the intestinal fungal microbiome, whereas matrine intervention partially preserves or restores fungal community balance. These results suggest that matrine exerts protective effects against DSS-induced colitis, at least in part, by modulating the composition and abundance distribution of the gut mycobiota, counteracting fungal dysbiosis and contributing to the reestablishment of a balanced intestinal microbial ecosystem.

To investigate the inter-kingdom interactions between gut bacteria and fungi, Spearman correlation analysis was performed based on the relative abundances of the top 10 bacterial and fungal genera (Supplementary Figure S2). The heatmap revealed distinct correlation patterns between bacterial and fungal taxa. Notably, beneficial bacterial genera, including *Acetatifactor*, *Roseburia*, *Eubacterium*, and *Muribaculum*, exhibited significant positive correlations (red squares, ^*^ *P* < 0.05, ^**^ *P* < 0.01) with several fungal genera such as *Fusarium*, *Malassezia*, and *Naganishia*. In contrast, the pathogenic bacterium *Escherichia* showed strong negative correlations (blue squares, ^*^ *P* < 0.05, ^**^ *P* < 0.01) with most fungal genera, particularly *Candida*, *Allocucurbitaria*, and *Penicillium*. Furthermore, *Akkermansia* displayed a complex interaction profile, showing negative correlations with *Candida* and *Penicillium* but a positive correlation with Aspergillus. These results suggest that matrine treatment may modulate the gut ecosystem by reshaping the co-occurrence networks between specific bacterial and fungal communities.

### 3.3 Matrine treatment effectively alleviated DSS-induced metabolic disturbances in the intestinal luminal metabolome of UC mice

Accumulating evidence indicates that gut microbiota dysbiosis in UC leads to significant alterations in host fecal metabolite profiles (Franzosa et al., 2019). Given the pivotal role of the gut microbiota in shaping host metabolism, the therapeutic effects of matrine in UC may extend beyond microbial modulation to include regulation of host metabolic pathways.

**FIGURE 7:**
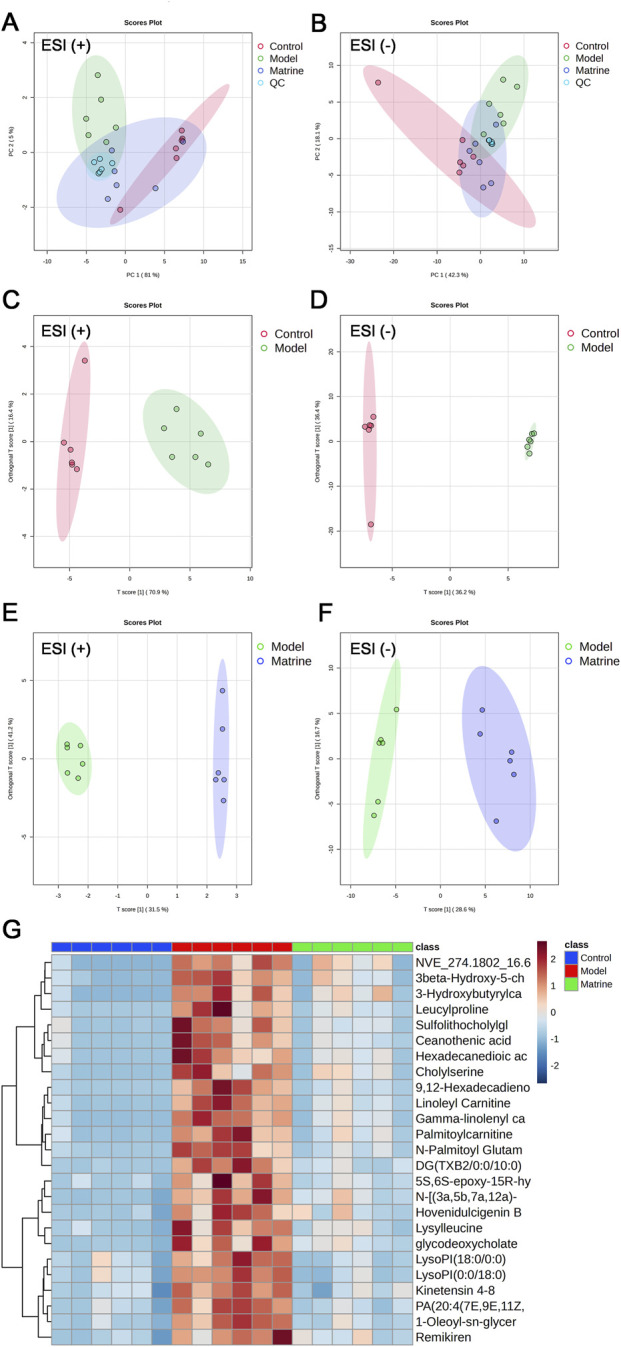
Multivariate analysis of untargeted metabolomics data from intestinal luminal contents. **(A,B)** PCA scores plots in positive **(A)** and negative **(B)** ionization modes. **(C–F)** OPLS-DA scores plots: Control vs. Model **(C,D)**; Model vs. Matrine **(E,F)**. Model parameters: **(C)** R^2^Y = 0.955, Q^2^ = 0.849; **(D)** R^2^Y = 0.995, Q^2^ = 0.842; **(E)** R^2^Y = 0.999, Q^2^ = 0.949; **(F)** R^2^Y = 0.966, Q^2^ = 0.777. **(G)** Heatmap of significantly altered metabolites across groups.

To investigate this, multivariate statistical analyses were performed on metabolomic profiles of intestinal luminal contents from UC mice across experimental groups to assess intra- and intergroup metabolic differences. PCA score plots—in both positive and negative ionization modes—revealed clear separation between the Control and Model groups (Figures 7A,B), confirming that DSS-induced colitis profoundly perturbed the intestinal luminal metabolome. Notably, the Matrine group exhibited a distinct metabolic trajectory, showing clear separation from the Model group and a trend toward clustering with the Control group, suggesting that matrine partially reverses the disease-associated metabolic disturbances and shifts the metabolic profile back toward homeostasis.

Orthogonal Partial Least Squares Discriminant Analysis (OPLS-DA) further validated these findings. In both ionization modes, the Control and Model groups were well-separated (Figures 7C,D), and model validation confirmed robustness without overfitting (Supplementary Figures S3A,C), indicating that DSS induction resulted in a significantly altered metabolic signature in the gut lumen. Similarly, OPLS-DA clearly distinguished the Matrine group from the Model group (Figures 7E,F), with rigorous model validation (Supplementary Figures S3B,D) supporting high predictive reliability and explanatory power for drug-induced metabolic changes.

Untargeted fecal metabolomics has emerged as a powerful tool for identifying biomarkers that aid in the diagnosis of UC, prediction of disease behavior, and assessment of therapeutic response, thereby facilitating the discovery of novel therapeutic targets (Vich et al., 2024). In this study, significantly altered metabolites between groups were selected based on the criteria: *P* < 0.05, variable importance in projection (VIP) > 1, and fold change (FC) > 2 or FC < 0.5. Particular attention was given to metabolites exhibiting a “reversal” effect—i.e., those dysregulated in the Model group but restored toward control levels by matrine treatment. The top 50 significantly altered metabolites in both positive and negative ionization modes were identified by matching their *m/z* values against MS Dial database and the HMDB, followed by stringent filtering for endogenous compounds.

Through this screening and annotation process, we identified 43 metabolites in intestinal luminal contents whose DSS-induced alterations were significantly reversed by matrine. These primarily belonged to three major chemical classes: steroids and steroid derivatives, fatty acyls, and carboxylic acids and derivatives (Table 1). A heatmap visualization confirmed that most of these metabolites were markedly elevated in the Model group compared to the Control group, while the Matrine group exhibited a clear trend toward normalization (Figure 7G), suggesting that matrine ameliorates UC-associated metabolic disturbances.

**TABLE 1:** Statistical analysis results of the main metabolites changed in gut content.

No.	Name	Class	HMDB	Rt (min)	Adduct	Formula	m/z	Control vs. model				Matrine vs. model			
								VIP	FC	p-value	Trend	VIP	FC	p-value	Trend
1	LysoPC (P-18:0/0:0)	Glycerophospholipids	HMDB0013122	14.43	M + H	C_26_H_54_NO_6_P	508.3762	1.06	0.03	0.0002	↓^***^	1.22	0.24	0.0120	↓^*^
2	Sulfolithocholylglycine	Steroids and steroid derivatives	HMDB0002639	7.09	M + H-H_2_O	C_26_H_43_NO_7_S	496.2733	1.11	0.08	0.0001	↓^***^	1.50	0.21	0.0015	↓^**^
3	Cholylserine	Steroids and steroid derivatives	HMDB0242388	8.98	M + H-H_2_O	C_27_H_45_NO_7_	478.3169	1.21	0.03	0.0000	↓^***^	1.29	0.34	0.0157	↓^*^
4	Ceanothenic acid	Prenol lipids	HMDB0036850	9.60	M + H	C_29_H_42_O_4_	455.3156	1.18	0.06	0.0000	↓^***^	1.48	0.21	0.0010	↓^***^
5	Hexadecanedioic acid mono-L-carnitine ester	Fatty acyls	HMDB0000712	10.20	M + H	C_23_H_43_NO_6_	430.3163	1.13	0.06	0.0000	↓^***^	1.40	0.26	0.0037	↓^**^
6	Linoleyl carnitine	Fatty acyls	HMDB0003073	13.58	M + H	C_25_H_45_NO_4_	424.3400	1.19	0.04	0.0000	↓^***^	1.53	0.23	0.0007	↓^***^
7	γ-linolenyl carnitine	Fatty acyls	HMDB0006318	12.99	M + H	C_25_H_43_NO_4_	422.3265	1.29	0.04	0.0000	↓^***^	1.56	0.25	0.0010	↓^***^
8	Palmitoylcarnitine	Organonitrogen compounds	HMDB0000284	14.24	M + H	C_23_H_45_NO_4_	400.3421	1.16	0.05	0.0000	↓^***^	1.45	0.26	0.0021	↓^**^
9	3β-hydroxy-5-cholestenoic acid	Steroids and steroid derivatives	HMDB0012453	8.81	M + H-H_2_O	C_27_H_44_O_3_	399.3263	1.25	0.07	0.0000	↓^***^	1.45	0.32	0.0060	↓^**^
10	4-cholesten-7α,12α,24-triol-3-one	Steroids and steroid derivatives	HMDB0062399	8.59	M + H-2H_2_O	C_27_H_44_O_4_	397.3113	1.21	0.06	0.0001	↓^***^	1.30	0.32	0.0105	↓^*^
11	9,12-Hexadecadienoylcarnitine	Fatty acyls	HMDB0013334	12.47	M + H	C_23_H_41_NO_4_	396.3108	1.21	0.02	0.0000	↓^***^	1.70	0.16	0.0002	↓^***^
12	N-Palmitoyl Glutamic acid	Carboxylic acids and derivatives	HMDB0241925	10.60	M + H	C_21_H_39_NO_5_	386.2901	1.32	0.03	0.0000	↓^***^	1.88	0.16	0.0003	↓^***^
13	3,5-Tetradecadiencarnitine	Fatty acyls	HMDB0013331	11.22	M + H	C_21_H_37_NO_4_	368.2795	1.03	0.03	0.0000	↓^***^	1.41	0.15	0.0009	↓^***^
14	Pregnenolone	Steroids and steroid derivatives	HMDB0000253	8.86	M + H	C_21_H_32_O_2_	317.2480	1.07	0.01	0.0001	↓^***^	1.24	0.21	0.0119	↓^*^
15	MG (16:0)	Glycerolipids	HMDB0072873	15.98	M + H-H_2_O	C_19_H_38_O_4_	313.2743	1.18	0.07	0.0000	↓^***^	1.15	0.50	0.0472	↓^*^
16	Dihydrosphingosine	Carboxylic acids and derivatives	HMDB0006780	12.14	M + H	C_18_H_39_NO_2_	302.3054	1.12	0.03	0.0000	↓^***^	1.00	0.39	0.0283	↓^*^
17	20α-Dihydroprogesterone	Steroids and steroid derivatives	HMDB0003069	8.86	M + H-H_2_O	C_21_H_32_O_2_	299.2375	1.02	0.01	0.0001	↓^***^	1.24	0.22	0.0118	↓^*^
18	NVE_274.1802_16.6	Phenol ethers	HMDB0341186	1.82	M + H	C_17_H_23_NO_2_	274.1802	1.37	0.05	0.0000	↓^***^	1.29	0.42	0.0232	↓^*^
19	γ-Glutamylleucine	Steroids and steroid derivatives	HMDB0062794	2.60	M + H	C_11_H_20_N_2_O_5_	261.1445	1.17	0.05	0.0001	↓^***^	1.34	0.28	0.0094	↓^**^
20	3-Hydroxybutyrylcarnitine	Fatty acyls	HMDB0013127	0.85	M + H	C_11_H_21_NO_5_	248.1492	1.30	0.05	0.0000	↓^***^	1.15	0.42	0.0209	↓^*^
21	PyroglutamylIsoleucine	Saturated hydrocarbons	HMDB0062790	4.35	M + H	C_11_H_18_N_2_O_4_	243.1344	1.18	0.03	0.0000	↓^***^	1.05	0.42	0.0398	↓^*^
22	Threonylleucine	Unsaturated hydrocarbons	HMDB0062786	1.51	M + H	C_10_H_20_N_2_O_4_	233.1510	1.37	0.03	0.0000	↓^***^	1.25	0.45	0.0411	↓^*^
23	Isoleucylvaline	Fatty acyls	HMDB0062784	2.56	M + H	C_11_H_22_N_2_O_3_	231.1707	1.17	0.01	0.0000	↓^***^	1.32	0.23	0.0069	↓^**^
24	Leucylproline	Organooxygen compounds	HMDB0062783	1.94	M + H	C_11_H_20_N_2_O_3_	229.1551	1.15	0.03	0.0000	↓^***^	1.41	0.21	0.0024	↓^**^
25	Prolylproline	Organic sulfuric acids and derivatives	HMDB0062775	0.85	M + H	C_10_H_16_N_2_O_3_	213.1249	1.22	0.05	0.0001	↓^***^	1.23	0.33	0.0107	↓^*^
26	Indole-3-pyruvic acid	Organic sulfuric acids and derivatives	HMDB0000682	0.87	M + Na	C_8_H_7_NO_4_S	204.0655	1.28	0.05	0.0001	↓^***^	1.44	0.30	0.0116	↓^*^
27	5S,6S-epoxy-15R-hydroxy-ETE	Organooxygen compounds	HMDB0062236	0.78	M + FA-H	C_9_H_20_O_5_	253.1293	1.58	0.14	0.0002	↓^***^	1.71	0.26	0.0006	↓^***^
28	Lysylleucine	Carboxylic acids and derivatives	HMDB0028955	1.49	M-H	C_12_H_25_N_3_O_3_	258.1823	1.68	0.14	0.0001	↓^***^	1.61	0.32	0.0016	↓^**^
29	Ala-Trp-Trp	Carboxylic acids and derivatives	HMDB0341128	7.02	M-H_2_O-H	C_25_H_27_N_5_O_4_	442.1879	2.26	0.05	0.0000	↓^***^	1.29	0.42	0.0236	↓^*^
30	10,11-Dihydro-12R-hydroxy-leukotriene E4	Fatty acyls	HMDB0012501	7.23	M-H	C_23_H_39_NO_6_S	456.2425	1.98	0.00	0.0000	↓^***^	1.11	0.33	0.0317	↓^*^
31	Chenodeoxycholylaspartic acid	Steroids and steroid derivatives	HMDB0242402	8.10	M-H	C_28_H_45_NO_7_	506.3123	1.70	0.09	0.0001	↓^***^	1.39	0.36	0.0057	↓^**^
32	Glycocholic acid 3-sulfate	Steroids and steroid derivatives	HMDB0240728	5.60	M-H_2_O-H	C_26_H_43_NO_9_S	526.2480	1.63	0.12	0.0001	↓^***^	1.65	0.34	0.0027	↓^**^
33	N-[(3a,5b,7a,12a)-3,12-dihydroxy-24-oxo-7-(sulfooxy)cholan-24-yl]-glycine	Steroids and steroid derivatives	HMDB0002640	6.10	M-H_2_O-H	C_26_H_43_NO_9_S	526.2475	1.80	0.11	0.0000	↓^***^	1.65	0.41	0.0012	↓^**^
34	Glycochenodeoxycholate-3-sulfate	Steroids and steroid derivatives	HMDB0002497	5.63	M-H	C_26_H_43_NO_8_S	528.2637	1.83	0.05	0.0000	↓^***^	1.60	0.25	0.0017	↓^**^
35	DG (TXB2/0:0/10:0)	Fatty acyls	HMDB0294484	13.34	M-H_2_O-H	C_33_H_58_O_9_	579.3897	2.18	0.05	0.0000	↓^***^	1.77	0.21	0.0001	↓^***^
36	Hovenidulcigenin B	Prenol lipids	HMDB0041547	12.61	M + FA-H	C_32_H_50_O_7_	591.3539	1.74	0.17	0.0001	↓^***^	1.77	0.42	0.0051	↓^**^
37	Cholestane-3,7,12,25-tetrol-3-glucuronide	Steroids and steroid derivatives	HMDB0010355	9.37	M-H	C_33_H_56_O_10_	611.3801	1.47	0.17	0.0005	↓^***^	1.68	0.25	0.0015	↓^**^
38	Remikiren	Carboxylic acids and derivatives	HMDB0014357	12.17	M-H	C_33_H_50_N_4_O_6_S	629.3378	1.49	0.24	0.0002	↓^***^	1.58	0.45	0.0026	↓^**^
39	PI	Glycerophospholipids	HMDB0242160	10.65	M-H	C_27_H_50_O_12_P	643.3100	1.92	0.16	0.0000	↓^***^	2.23	0.25	0.0000	↓^***^
40	LysoPI(0:0/18:0)	Glycerophospholipids	HMDB0061704	9.30	M-H	C_27_H_53_O_12_P	645.3257	1.60	0.30	0.0002	↓^***^	2.19	0.24	0.0000	↓^***^
41	LysoPI(18:0/0:0)	Glycerophospholipids	HMDB0240261	9.87	M-H	C_27_H_53_O_12_P	645.3257	1.50	0.28	0.0006	↓^***^	2.10	0.24	0.0000	↓^***^
42	PA (8:0_20:4-3OH)	Glycerophospholipids	HMDB0266628	11.06	M-H	C_31_H_53_O_11_P	677.3308	1.69	0.23	0.0001	↓^***^	2.09	0.28	0.0000	↓^***^
43	Kinetensin 4-8	Carboxylic acids and derivatives	HMDB0012987	11.44	M-H_2_O-H	C_35_H_46_N_10_O_7_	699.3367	1.58	0.34	0.0002	↓^***^	1.78	0.40	0.0001	↓^***^

Notably, a substantial proportion of the steroid-related metabolites modulated by matrine comprised are cholesterol-derived BAs and their conjugated or microbially transformed derivatives. These included sulfolithocholylglycine, cholylserine, 3β-hydroxy-5-cholestenoic acid, 4-cholesten-7α,12α,24-triol-3-one, pregnenolone, 20α-dihydroprogesterone, γ-glutamylleucine, chenodeoxycholylaspartic acid, glycocholic acid 3-sulfate, N-[(3α,5β,7α,12α)-3,12-dihydroxy-24-oxo-7-(sulfooxy)cholan-24-yl]-glycine, glycodeoxycholate sulfate, glycochenodeoxycholate-3-sulfate, and N-[(3α,5β,7α)-3-hydroxy-24-oxo-7-(sulfooxy)cholan-24-yl]-glycine. Clinical and preclinical studies have established that BAs homeostasis is tightly linked to UC severity: patients with UC typically exhibit elevated levels of primary BAs and reduced secondary BAs in feces (Stefanie Sommersberger, 2023). In UC models, gut microbiota dysbiosis impairs the microbial conversion of primary to secondary BAs—a process critical for maintaining intestinal immune tolerance. The resulting deficiency in secondary BAs exacerbates mucosal inflammation (Lee et al., 2024; Sinha et al., 2020). Moreover, clinical evidence shows that disease activity correlates with increased fecal dipeptides and taurine-conjugated BAs, alongside decreased glycine-conjugated forms (Schirmer et al., 2024). Our metabolomic analyses demonstrate that matrine effectively reverses DSS-induced aberrant accumulation of specific BAs species, thereby restoring BAs homeostasis.

Another key finding is the modulation of 10,11-dihydro-12R-hydroxy-leukotriene E4, a reduced hydroxylated metabolite of leukotriene E4 (LTE4) derived from the arachidonic acid inflammatory pathway. LTE4 is a well-established biomarker of intestinal inflammation, as its production is upregulated during active UC due to heightened inflammatory signaling (Stanke-Labesque et al., 2008; Wang et al., 2024). Consistent with this, we observed elevated levels of 10,11-dihydro-12R-hydroxy-leukotriene E4 in the luminal contents of UC mice, which were significantly suppressed following matrine treatment—indicating inhibition of pro-inflammatory eicosanoid pathways.

Furthermore, several differentially regulated metabolites are directly linked to colonic epithelial energy metabolism, including linoleyl carnitine, γ-linolenyl carnitine, palmitoylcarnitine, 3-hydroxybutyrylcarnitine, 9,12-hexadecadienoylcarnitine, and 3,5-tetradecadiencarnitine—collectively classified as acylcarnitines. Acylcarnitines serve as recognized biomarkers of IBD activity. Under healthy conditions, colonocytes preferentially utilize SCFAs, particularly butyrate, as their primary energy source. However, in UC, dysbiosis-driven SCFA deficiency and mitochondrial dysfunction impair β-oxidation of medium- and long-chain fatty acids, leading to compensatory accumulation of acylcarnitines. This buildup not only reflects metabolic stress but also actively suppresses epithelial barrier repair (Ho and Theiss, 2022; Jackson and Theiss, 2020; Novak and Mollen, 2015). Critically, our intestinal luminal metabolomics data show that matrine significantly reduces the accumulation of these acylcarnitines, suggesting that it alleviates UC by restoring mitochondrial energy metabolism and supporting epithelial integrity.

### 3.4 Correlation analysis between gut microbiota and differentially abundant metabolites

**FIGURE 8:**
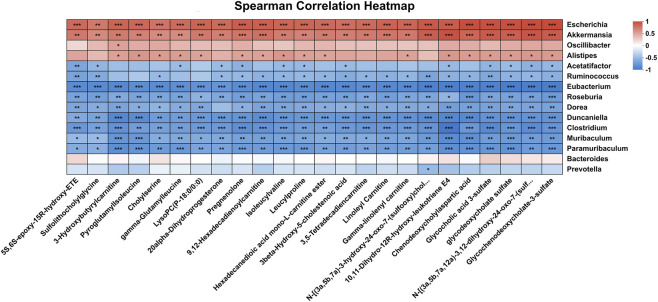
Microbe-metabolite correlation analysis. Spearman’s rank correlation heatmaps illustrating the associations between gut microbial genera and fecal metabolites in UC mice. The color intensity of each square represents the Spearman correlation coefficient (r), ranging from negative (blue) to positive (red) correlations. The significance of the correlations is indicated by asterisks ^*^ *P* < 0.05, ^**^ *P* < 0.01, ^***^ *P* < 0.001. Only correlations with |r| > 0.6 and *P* < 0.05 were displayed to ensure reliability.

Our prior findings demonstrated that matrine effectively alleviates DSS-induced UC in mice by modulating both gut microbial composition and the host intestinal luminal metabolome. To further elucidate the underlying mechanisms, Spearman correlation analysis was performed at the genus level to systematically investigate the associations between gut bacterial taxa and differentially abundant metabolites. As shown in Figure 8, several classes of dysregulated metabolites—including cholesterol-derived steroid compounds (e.g., sulfolithocholylglycine, cholylserine, 3β-hydroxy-5-cholestenoic acid, 4-cholesten-7α,12α,24-triol-3-one, pregnenolone, 20α-dihydroprogesterone, γ-glutamylleucine, chenodeoxycholylaspartic acid, glycocholic acid 3-sulfate, glycodeoxycholate sulfate, and glycochenodeoxycholate-3-sulfate), inflammatory mediators (e.g., 10,11-dihydro-12R-hydroxy-leukotriene E4 and 5S,6S-epoxy-15R-hydroxy-ETE), and acylcarnitines (e.g., linoleyl carnitine, γ-linolenyl carnitine, palmitoylcarnitine, 3-hydroxybutyrylcarnitine, 9,12-hexadecadienoylcarnitine, and 3,5-tetradecadiencarnitine)—were all significantly positively correlated (*P* < 0.05) with the opportunistic pathogen *Escherichia*. Conversely, these same metabolites exhibited significant negative correlations (*P* < 0.05) with several potentially beneficial genera, including *Eubacterium*, *Duncaniella*, *Clostridium*, *Muribaculum*, and *Paramuribaculum*. These findings imply that the accumulation of detrimental metabolites is closely linked to the overgrowth of pro-inflammatory bacteria, while the depletion of protective microbes may impair the host’s capacity to counteract such metabolic disturbances.

Notably, previous studies have reported a marked reduction in *Muribaculum* abundance in DSS-induced UC mice (Wang et al., 2022), and its supplementation significantly enhances fecal SCFAs levels (Gu et al., 2022; Yamane et al., 2021). *Paramuribaculum*, a phylogenetically related genus, shares anti-inflammatory properties with *Muribaculum* and is also depleted in UC patients, showing strong associations with SCFA-related metabolic pathways (Fang et al., 2023; Qiu et al., 2025). We therefore propose that *Muribaculum* and *Paramuribaculum* may act synergistically to regulate the levels of inflammation-associated metabolites and energy metabolism intermediates—such as acylcarnitines—in the gut lumen. Consistent with prior observations, *Escherichia* abundance has been positively associated with UC disease activity, whereas *Eubacterium*, *Duncaniella*, and *Clostridium* are known to suppress intestinal inflammation and promote the production of beneficial metabolites like SCFAs. Correlation analysis revealed that metabolites significantly downregulated by matrine treatment are predominantly positively correlated with harmful taxa like *Escherichia* and negatively correlated with these beneficial genera. Further implicating microbial–metabolite interactions in the therapeutic effects of matrine. It is important to acknowledge that a direct measurement of SCFA levels was not performed in the current study. While our metagenomic analysis and reference to existing literature suggest a correlation between the enriched microbiota and SCFA production, the lack of direct metabolite quantification represents a limitation. Collectively, these findings implicate microbiota–metabolite interactions as a central component of the therapeutic effects of matrine, whereby coordinated modulation of microbial composition and metabolic output leads to the suppression of inflammatory mediators and aberrant lipid metabolites, preservation of intestinal barrier integrity, and attenuation of mucosal inflammation.

## 4 Conclusion

In conclusion, integrated multi-omics analysis revealed that matrine exerted protective effects in DSS-induced colitis by concurrently restoring gut bacterial and fungal homeostasis and correcting intestinal luminal metabolic dysregulation. Matrine ameliorated clinical and histological manifestations of UC, suppressed pro-inflammatory pathways, normalized bile acid and acylcarnitine metabolism, and enhanced intestinal barrier integrity. Notably, its therapeutic efficacy was closely associated with the enrichment of SCFA-producing symbionts (e.g., *Muribaculum*, *Paramuribaculum*) and suppression of pathobionts such as *Escherichia*, which strongly correlated with beneficial shifts in the metabolome. These findings position matrine as a promising microbiota- and metabolism-targeted natural agent for IBD therapy, offering a mechanistic foundation for its clinical development.

## Data Availability

The datasets presented in this study can be found in online repositories. The names of the repository/repositories and accession number(s) can be found in the article/Supplementary Material.
