# Supplementary material for: Integrated metabolomics and gut microbiota analyses reveal the protective effects of matrine in ulcerative colitis
Source: Front Chem. 2026 May 13;14:1826894. doi: 10.3389/fchem.2026.1826894 (PMC13213417; doi:10.3389/fchem.2026.1826894)
Supplement: Supplementary file 1 [file DataSheet1.docx]

**Supplementary Material**

**1 Gut microbiota shotgun metagenomic sequencing analysis**

0.2g of intestinal luminal contents material was used to extract total genomic DNA with the FastPure^®^ Stool DNA Isolation Kit (Magnetic Bead) (MJYH Biotech, Shanghai, China) according to manufacturer's instructions. Concentration and purity of extracted DNA was determined with Synergy HTX (Biotek, USA) and NanoDrop2000 (Thermo Scientific Inc., USA), respectively. DNA quality was checked on 1% agarose gel.

DNA extract was fragmented to an average size of about 350 bp using Covaris M220 (Gene Company Limited, China) for paired-end library construction. Paired-end library was constructed using NEXTFLEX^®^ Rapid DNA-Seq (Bioo Scientific, Austin, TX, USA). Shotgun metagenomic sequencing was performed on DNBSEQ-T7 platform at Majorbio Bio-Pharm Technology Co., Ltd. (Shanghai, China) using DNBSEQ-T7RS Reagent Kit (FCL PE150) version 3.0, according to the manufacturer's instructions. The data were analyzed on the free online platform of Majorbio Cloud Platform (www.majorbio.com). Briefly, the raw sequencing reads were trimmed of adapters, and low-quality reads (length<50 bp or with average quality value<20) were removed by fastp (<https://github.com/OpenGene/fastp>, version 0.23.0). Reads were aligned to the mice genome by BWA (<http://bio-bwa.sourceforge.net>, version 0.7.17) and any hit associated with the reads and their mated reads were removed. The quality-filtered data were assembled using MEGAHIT (<https://github.com/voutcn/megahit>, version 1.2.9). Contigs with a length ≥300 bp were selected as the final assembling result.Open reading frames (ORFs) from each assembled contigs were predicted using Prodigal" (<https://github.com/hyattpd/Prodigal,version2.6.3>) and a length ≥100 bp ORFs were retrieved. A non-redundant gene catalog was constructed using CD-HIT14 (http://wei zhongli-lab.org/cd-hit/, version 4.6.1) with 90sequence identity and 90% coverage. Gene abundance for a certain sample was eatimated by SOAPaligner (<https://github.com/ShujiaHuang/SOAPaligner>, version soap2.21release) with 95% identity.

**2 Internal Transcribed Spacer (ITS) sequencing analysis**

Total microbial genomic DNA was extracted from intestinal luminal contents of mice using the FastPure Stool DNA Isolation Kit (MJYH, shanghai, China) according to manufacturer's instructions. The quality and concentration of DNA were determined by 1.0% agarose gel electrophoresis and a NanoDrop^®^ND-2000 spectrophotometer (Thermo Scientific Inc., USA) and kept at 80 °C prior to further use. The fungal ITS1–ITS2 region was amplified with the primer pair ITS1F (5’-CTTGGTCATTTAGAGGAAGTAA-3’) and ITS2R (5’-GCTGCGTTCTTCATCGATGC-3’) using a T100 Thermal Cycler (Bio-Rad, USA). The PCR reaction mixture including 10 μL 2×Phanta Max Master Mix, 0.8 μL each primer (5 μM), 10 ng of template DNA, and ddH_2_O to a final volume of 20 μL. PCR amplification cycling conditions were as follows: initial denaturation at 95 °C for 3 min, followed by 35 cycles of denaturing at 95 °C for 30 s, annealing at 55 °C for 30 s and extension at 72 °C for 45 s, and single extension at 72 °C for 10 min, and end at 10 °C. All samples were amplified in triplicate. The PCR product was extracted from 2% agarose gel and purified. Then quantified using Synergy HTX (Biotek, USA). Purified amplicons were pooled in equimolar amounts and paired-end sequenced on an IIlumina NextSeq 2000 PE300 platform (Ilumina, San Diego, USA) according to the standard protocolsby Majorbio Bio-Pharm Technology Co. Ltd. (Shanghai, China).

Paired-end raw sequencing reads were subjected to quality control using fastp (<https://github.com/OpenGene/fastp>, version 0.23.4) and subsequently merged using FLASH (<http://ccb.jhu.edu/software/FLASH/>, version 1.2.11) with the following parameters: (1) Bases at the 3’ ends with Phred quality scores < 20 were trimmed; a sliding window of 50 bp was applied, and if the average quality within the window fell below 20, all bases from the start of the window onward were removed. Reads shorter than 50 bp after trimming, as well as those containing more than five ambiguous (N) bases, were discarded. (2) Paired-end reads were merged into single contiguous sequences based on their overlapping regions, with a minimum overlap length of 10 bp. (3) Merged sequences were retained only if the mismatch rate within the overlap region did not exceed 0.2. (4) Sample demultiplexing was performed based on barcodes and primers located at the 5’ and 3’ ends of each read; barcode mismatches were not permitted (0 allowed), while up to two primer mismatches were tolerated.

The resulting high-quality merged sequences were denoised using the DADA2 plugin within the QIIME 2 (Quantitative Insights Into Microbial Ecology) pipeline under default parameters. This process generated amplicon sequence variants (ASVs), which represent exact biological sequences without clustering. Taxonomic assignment of ASVs was performed using the classify-sklearn (Naive Bayes) classifier in QIIME 2 against the UNITE v9.0 reference database for fungal ITS sequences (confidence threshold = 70%). Prior to downstream analysis, ASVs annotated as originating from chloroplasts or mitochondria were removed across all samples to eliminate potential contamination from host or plant DNA.

Alpha diversity indices (e.g., observed Sobs, Ace index) and beta diversity metrics were computed using Mothur (version 1.30.2). Visualization was implemented in R (version 3.3.1). Community composition was further visualized using stacked bar plots and heatmaps. Statistical comparisons of ASV abundances between groups were conducted using the Kruskal–Wallis test for multi-group analyses or the Wilcoxon rank-sum test for pairwise comparisons, with significance determined based on mean differential abundance. The linear discriminant analysis (LDA) effect size (LEfSe) (<http://huttenhower.sph.harvard.edu/LEfSe>) was performed to identify the significantly abundant taxa (phylum to genera) of fungi among the different groups (LDA score>2, P<0.05).

**3 Untargeted Metabolomic Analysis of Intestinal Luminal Contents**

Intestinal luminal contents were subjected to untargeted metabolomic profiling as follows. Samples were lyophilized and homogenized into fine powder. For metabolite extraction, 50 mg of powder was suspended in ice-cold acetonitrile–methanol (1:1, v/v) to a final concentration of 50 mg/mL. The mixture was vortexed and sonicated for 15 min in an ice-water bath, followed by centrifugation at 14,000 rpm at 4 °C for 10 min. A 100-μL aliquot of the supernatant was transferred to a new tube and dried under a gentle stream of nitrogen. The residue was reconstituted in 100 μL of 50% aqueous acetonitrile, vortexed for 3 min, and sonicated for an additional 15 min. After a second centrifugation (14,000 rpm, 4 °C, 10 min), the supernatant was transferred to autosampler vials for analysis. To monitor system stability and data quality, a pooled quality control (QC) sample was prepared by mixing equal volumes of all processed samples and injected repeatedly throughout the analytical batch.

Chromatographic separation was performed on an Agilent 1290 II UHPLC system (Agilent Technologies, Santa Clara, CA, USA) equipped with a ZORBAX Eclipse XDB-C18 column (2.1 × 100 mm, 1.8 μm). The mobile phase consisted of (A) 0.1% formic acid in water and (B) acetonitrile. The gradient program was as follows: 0–10 min, 5–60% B; 10–13 min, 60–95% B; 13–17 min, 95% B; 17–17.1 min, 95–5% B; 17.1–20 min, 5% B. The flow rate was maintained at 0.3 mL/min, column temperature at 30 °C, and injection volume at 2 μL.

Mass spectrometry detection was carried out on an AB SCIEX ZenoTOF™ 7600 high-resolution mass spectrometer (AB Sciex, Framingham, MA, USA) coupled to the UHPLC system via an electrospray ionization (ESI) source. Both positive and negative ionization modes were employed in separate runs.

In positive ion mode, the following parameters were used: nebulizer gas (GS1) = 50 psi, heater gas (GS2) = 50 psi, curtain gas = 35 psi, collision gas = 8 (arbitrary units), source temperature = 500 °C, capillary voltage = 3.0 kV, declustering potential (DP) = +50 V, and spray voltage = 5500 V. Full-scan TOF MS data were acquired over m/z 100–1000 with a cycle time of 0.15 s, dynamic background subtraction (DBS) enabled, and collision energy (CE) set to 12 V. Information-dependent acquisition (IDA) triggered MS/MS scans (m/z 50–1000) with DP = +50 V, CE = 35 V, and collision energy spread (CES) = 15 V; accumulation time = 0.05 s.

In negative ion mode, source conditions were identical except for the following: DP = –80 V, CE = –10 V for MS scans, and CE = –45 V for MS/MS scans; all other voltages and gas settings remained unchanged.

Raw data were processed using Progenesis QI (Waters Corporation) for peak detection, alignment, and metabolite annotation. Putative metabolites were subsequently identified through spectral matching against MS-DIAL and the Human Metabolome Database (HMDB).

**4 Supplementary Tables and Figures**

Table S1 Scoring criteria for Disease Activity Index (DAI)

| Score | Weight Loss (%) | Stool Consistency | Fecal Bleeding |
| --- | --- | --- | --- |
| 0 | ＜0 | Normal | No bleeding |
| 1 | 0-5 | Soft stool | Mild bleeding |
| 2 | 5-10 | Loose stool | Mild bleeding |
| 3 | 10-15 | Diarrhea | Moderate bleeding |
| 4 | ＞15 | Severe diarrhea | Severe bleeding |

Table S2 Histopathological scoring criteria for colonic tissue

| Score | Inflammatory Infiltration | Glandular Damage | Lesion Area |
| --- | --- | --- | --- |
| 0 | No inflammatory cell infiltration | Intact crypts, no damage | None |
| 1 | Infiltration around crypts | Crypt loss <1/3 (mild local damage) | 0-25% |
| 2 | Infiltration in mucosal muscularis | Crypt loss >1/3 but ≤1/2 (mild damage) | 26-50% |
| 3 | Diffuse infiltration in mucosal muscularis | Crypt loss >1/2 but ≤2/3 (moderate damage) | 51-75% |
| 4 | Infiltration into submucosa, mucosal hyperplasia | Complete crypt loss | 76-100% |


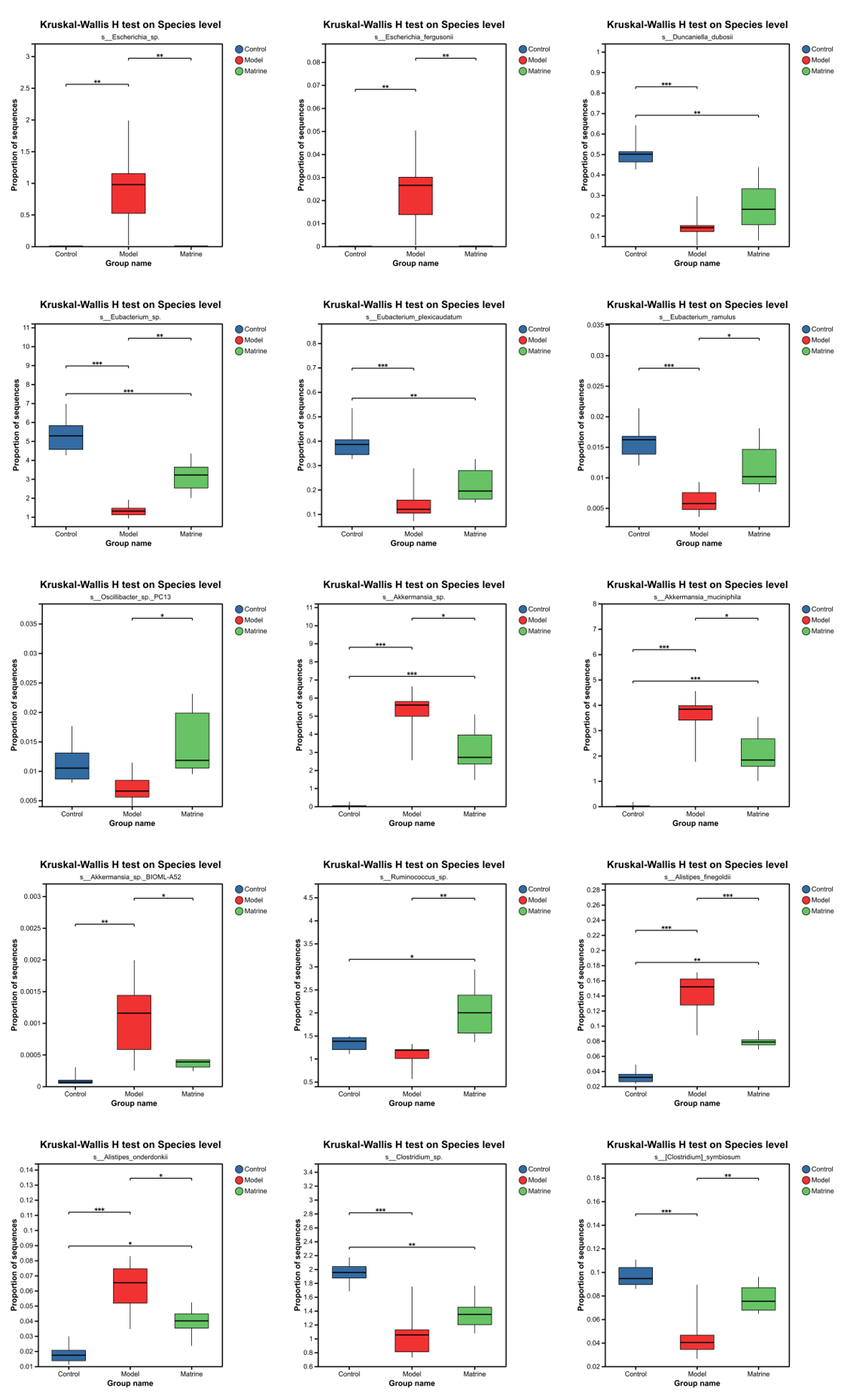


**FIGURE S1** Relative abundance of significantly different bacterial species across Control, Model, and Matrine-treated groups. The relative abundance of each species was compared using the Kruskal-Wallis H test. Statistical significance is denoted as ^*^*P* < 0.05, ^**^*P* < 0.01, and ^***^*P* < 0.001. Box plots indicate the median, interquartile range (IQR), and data distribution for each group.


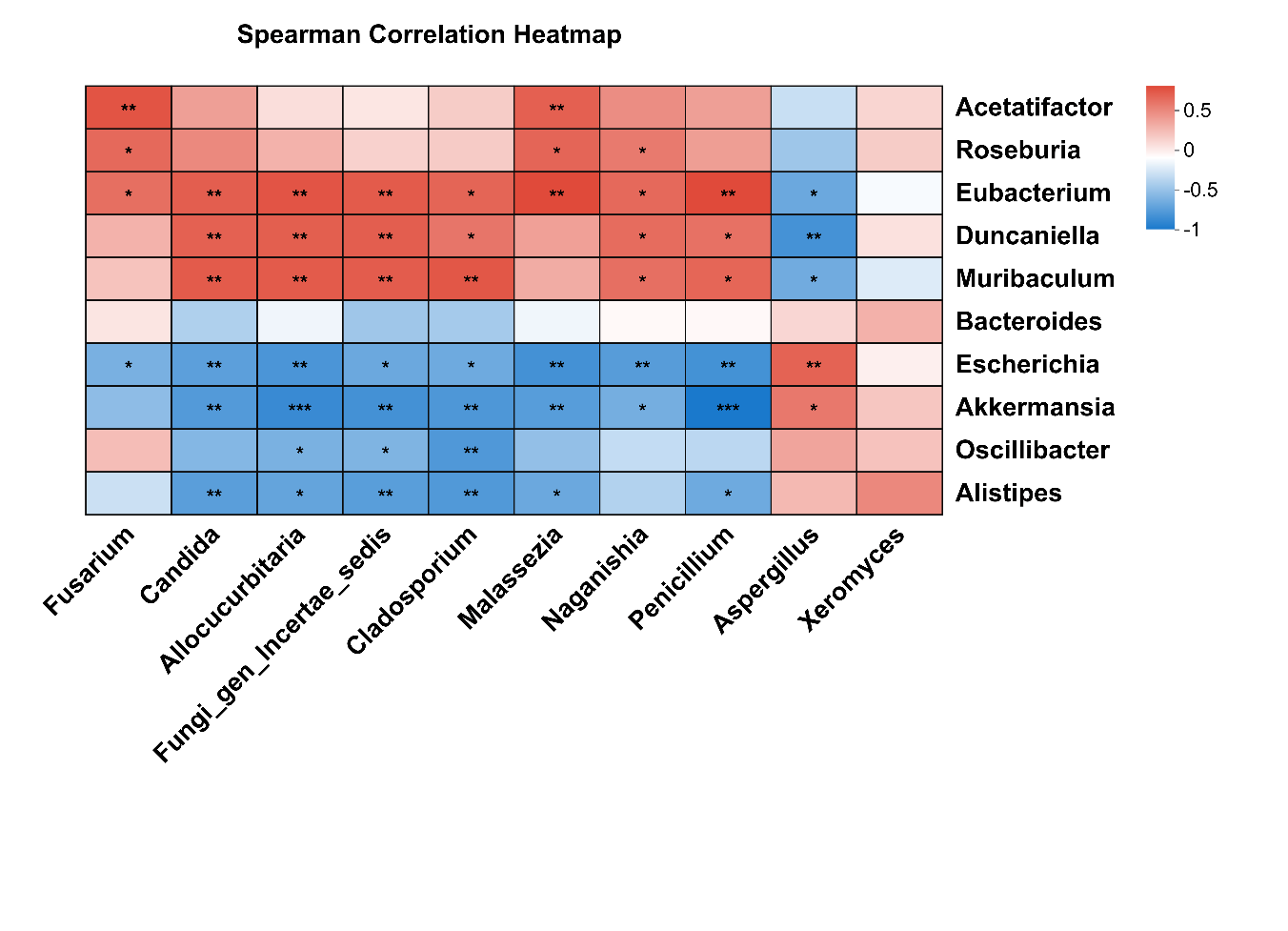


**FIGURE S2** Cross-kingdom correlation analysis. Spearman’s rank correlation heatmaps illustrating the associations between bacterial and fungal genera in UC mice. The color intensity of each square represents the Spearman correlation coefficient (r), ranging from negative (blue) to positive (red) correlations. The significance of the correlations is indicated by asterisks ^*^*P* < 0.05, ^**^*P* < 0.01, ^***^*P* < 0.001. Only correlations with |r| > 0.6 and *P* < 0.05 were displayed to ensure reliability.

**FIGURE S3** Orthogonal Partial Least Squares–Discriminant Analysis (OPLS-DA) models in positive (A, B) and negative (C, D) ionization modes assessing the effect of matrine on the metabolic profiles of intestinal luminal contents in DSS-induced ulcerative colitis (UC) mice. Panels A and C show the comparison between Control and Model groups; panels B and D compare Model versus Matrine-treated groups. Model parameters: (A) R²Y = 0.955, Q² = 0.849; (B) R²Y = 0.995, Q² = 0.842; (C) R²Y = 0.999, Q² = 0.949; (D) R²Y = 0.966, Q² = 0.777.
